# Vortioxetine Improved Depressive State In Parkinson’s Disease

**DOI:** 10.7759/cureus.15750

**Published:** 2021-06-18

**Authors:** Reiji Yoshimura, Atsuko Ikenouchi, Naomichi Okamoto, Yuki Konishi

**Affiliations:** 1 Psychiatry, University of Occupational and Environmental Health, Kitakyushu, JPN

**Keywords:** vortioxetine, depression, parkinson’s disease, case report, levodopa

## Abstract

We report the case of a 67-year-old Japanese Parkinson’s disease (PD) patient with depression who was successfully treated with vortioxetine. The patient’s PD was being treated with levodopa (100 mg/day). As improvement was noted in PD symptoms, levodopa was continued. Subsequently, she was referred to our department due to worsening of her depressive symptoms, including depressed mood, anhedonia, decreased energy, decreased concentration, decreased motivation, insomnia, hopelessness, and worthlessness. The patient did not respond to paroxetine and escitalopram. Finally, vortioxetine improved her depressive state without worsening PD.

## Introduction

Parkinson’s disease (PD) is one of the most common and severe movement disorders globally, affecting about 1% of adults older than 60 years [1]. PD results from the loss of dopamine neurons in the substantia nigra, and its cause is not yet elucidated in most individuals [1]. Depression is common among PD patients. It is generally accepted that clinically significant depressive disturbances occur in 40-50% of patients with PD and affect various clinical aspects of the disease [2]. In addition to causing inherent mood distress, depressive disorders negatively impact patients’ quality of life, motor and cognitive deficits, and functional disability. For patients, these neuropsychiatric symptoms are often more problematic and distressing than the motor symptoms of PD. A recent network meta-analysis demonstrated that selective serotonin reuptake inhibitors (SSRIs), serotonin noradrenaline reuptake inhibitors (SNRIs), and tricyclic antidepressants (TCAs) were efficacious against depression among PD patients and improved their activities of daily living and motor functions [3-6]. However, adverse effects have been reported including extrapyramidal symptoms [7,8]. Here, we report the case of a PD patient with depressive disorder due to another medical condition based on the Diagnostic and Statistical Manual of Mental Disorders Fifth Edition (DSM-5), who was successfully treated with vortioxetine but was unresponsive to paroxetine and escitalopram without worsening the extrapyramidal symptoms of PD.

## Case presentation

A 67-year-old Japanese woman was referred to a general hospital and was diagnosed with PD after examination by a neurologist. Her clinical presentation included masking of facial expressions, decreased eye blink rate, bradykinesia, and cogwheel rigidity. She was treated with levodopa (100 mg/day). As her PD improved, levodopa was continued. Subsequently, she was referred to our department at the University of Occupational and Environmental Health, Japan, due to worsening of her depressive symptoms a month before, including depressed mood, anhedonia, decreased energy, decreased concentration, decreased motivation, insomnia, hopelessness, and worthlessness without any particular life events or stressors. Moreover, she had never experienced previous depressive episodes. In particular, her anhedonia and decreased energy, concentration, and motivation were severe. Her Mini-Mental State Examination score was 30/30, indicating no specific cognitive impairment. Magnetic resonance imaging revealed no abnormality. Her endocrinologic examination was also normal. She was diagnosed with depressive disorder due to another medical condition according to the DSM-5. Her Hamilton Rating Scale for Depression (HAMD) score was 26. Her depressive symptoms did not improve with paroxetine (40 mg/day) for four weeks or venlafaxine (100 mg/day) for four weeks. The mean HAMD score was 24. Venlafaxine was switched to escitalopram, with the dose increased to 20 mg/day. On switching the medication, her depressive symptoms slightly improved. The HAMD score reduced to 19 after six weeks of escitalopram administration. Further improvement was observed after escitalopram treatment for six weeks. As her depressive symptoms did not respond to treatment with typical SSRIs or SNRIs, she was started on vortioxetine (10 mg/day). Vortioxetine dosage was increased to 20 mg/day, and escitalopram was tapered off. Four weeks after the initiation of vortioxetine treatment, her depressive state gradually improved, and her HAMD score decreased to 10. Her depressive state went into remission six weeks after treatment with vortioxetine, and her HAMD score decreased to 4. During the treatment period, levodopa was sustained. Currently, she is doing well and continues to take levodopa (100 mg/day) and vortioxetine (20 mg/day). Worsening PD and depressive symptoms have not been observed.

## Discussion

In the present case, vortioxetine successfully ameliorated the depressive symptoms of the PD patient who was resistant to paroxetine and escitalopram treatment. SSRIs, SNRIs, and TCAs have been the standard treatment for depression in patients with PD. Hence, while patients may not respond to one group of medications, they may respond to a different medication. However, several meta-analyses have suggested that the current evidence on the efficacy of TCAs and SSRIs in treating psychiatric symptoms is inconclusive [4-6,9-12]. TCAs are difficult to prescribe for presenile depression with PD because of their anticholinergic effects. Antidepressant-induced parkinsonism has been reported in patients treated with escitalopram and trazodone [13,14]. In contrast, another study suggested that SSRIs did not significantly worsen extrapyramidal symptomatology [15]. However, this needs further investigation. In the present case, SSRIs and SNRIs, including paroxetine, venlafaxine, escitalopram, and vortioxetine, did not worsen the patient’s parkinsonism. The exact mechanism of action of vortioxetine in improving the depressive symptoms of our patient remains unknown. A novel profile of vortioxetine for various serotonin receptors, including 5-HT_1A_, 5-HT_1B_, 5-HT_1D_, 5-HT_3_, and 5-HT_7_, as well as serotonin transporters (5-HTT), is likely related to its clinical efficacy for ameliorating the depressive symptoms in PD patients. In short, vortioxetine is a 5-HT_3_, 5-HT_7_, and 5-HT_1D_ receptor antagonist, 5-HT_1B_ receptor partial agonist, 5-HT_1A_ receptor agonist, and 5-HTT inhibitor [16]. It has been reported that the blockade of 5-HT_6_ and 5-HT_7_ receptors increase the release of dopamine [17]. Thus, it is plausible that the 5-HT_7_ antagonistic effects of vortioxetine might also increase the release of dopamine. Another report suggested the enhanced release of dopamine, norepinephrine, acetylcholine, and histamine via several 5-HT_1A_ and 5-HT_1B_ receptors [18]. A recent post-marketing study in the world pharmacovigilance database [19] reported a potentially harmful association between movement disorders and the use of antidepressants, including mirtazapine, vortioxetine, amoxapine, fluvoxamine, citalopram, paroxetine, duloxetine, bupropion, clomipramine, escitalopram, fluoxetine, mianserin, sertraline, venlafaxine, and vilazodone. Another study demonstrated an association between SSRI use and apathy in PD patients [20]. Clinicians must be aware of these adverse effects and should continue to monitor their patients undergoing treatment. On the other hand, the multimodal antidepressant vortioxetine and the dual-action SNRIs, including venlafaxine, desvenlafaxine, and duloxetine, are considered to be good options for PD patients with depression [21].

## Conclusions

We reported the case of a presenile female with PD suffering from depressive symptoms. Adding vortioxetine to her ongoing levodopa regimen resulted in the complete remission of her depressive symptoms without worsening PD, which was not contradictory to the recent expert opinion. This case indicated that vortioxetine is a viable treatment option for depression with severe anhedonia and psychomotor retardation in PD patients.
